# Formerly bile-farmed bears as a model of accelerated ageing

**DOI:** 10.1038/s41598-023-36447-z

**Published:** 2023-06-15

**Authors:** Szilvia K. Kalogeropoulu, Hanna Rauch-Schmücking, Emily J. Lloyd, Peter Stenvinkel, Paul G. Shiels, Richard J. Johnson, Ole Fröbert, Irene Redtenbacher, Iwan A. Burgener, Johanna Painer-Gigler

**Affiliations:** 1 Department of Interdisciplinary Life Sciences, Research Institute of Wildlife Ecology, University of Veterinary Medicine, 1160 Vienna, Austria; 2 BEAR SANCTUARY Ninh Binh, FOUR PAWS Viet, Ninh Binh, 43000 Vietnam; 3 Department of Renal Medicine M99, Karolinska, University Hospital, 141 86 Stockholm, Sweden; 4 Davidson Bld, School of Molecular Biosciences, University of Glasgow, Glasgow, GB UK; 5 Division of Renal Diseases, University of Colorado Anschutz Medical Campus, Aurora, CO USA; 6 Department of Cardiology, Faculty of Health, Örebro University, Örebro, Sweden; 7 Steno Diabetes Center Aarhus, Aarhus University Hospital, Aarhus, Denmark; 8 Department of Clinical Medicine, Faculty of Health, Aarhus University, Aarhus, Denmark; 9 Department of Clinical Pharmacology, Aarhus University Hospital, Aarhus, Denmark; 10 Four Paws International, Vienna, Austria; 11 Division of Small Animal Internal Medicine, Department for Companion Animals and Horses, University of Veterinary Medicine, 1210 Vienna, Austria

**Keywords:** Physiology, Diseases, Gastroenterology, Nephrology, Pathogenesis, Risk factors

## Abstract

Bear bile-farming is common in East and Southeast Asia and this farming practice often results in irreversible health outcomes for the animals. We studied long-term effects of chronic bacterial and sterile hepatobiliary inflammation in 42 Asiatic black bears (*Ursus thibetanus*) rescued from Vietnamese bile farms. The bears were examined under anesthesia at least twice as part of essential medical interventions. All bears were diagnosed with chronic low-grade sterile or bacterial hepatobiliary inflammation along with pathologies from other systems. Our main finding was that the chronic low-grade inflammatory environment associated with bile extraction in conjunction with the suboptimal living conditions on the farms promoted and accelerated the development of age-related pathologies such as chronic kidney disease, obese sarcopenia, cardiovascular remodeling, and degenerative joint disease. Through a biomimetic approach, we identified similarities with inflammation related to premature aging in humans and found significant deviations from the healthy ursid phenotype. The pathological parallels with inflammageing and immuno-senescence induced conditions in humans suggest that bile-farmed bears may serve as animal models to investigate pathophysiology and deleterious effects of lifestyle-related diseases.

## Introduction

Biomimetics (‘inspiration by nature’) is the study of nature and natural phenomena to understand the principles of underlying mechanisms, to obtain ideas from nature, and to apply concepts that may benefit science, engineering, and medicine^1^. In the context of medicine, biomimetic studies of non-laboratory wild animals are useful for identifying mechanisms that protect or increase susceptibility to age-associated burden of lifestyle diseases^2^. The bio-inspired approach could provide novel means for developing treatments and products intended for medical use^3^.

Across East and Southeast Asia, > 17,000 Asiatic black bears (*Ursus thibetanus*), Malayan sun bears (*Helarctos malayanus*) and brown bears (*Ursus arctos*) are farmed for their bile to meet the consumer demand for traditional medicines^4^. Most bile-farmed bears are kept in small metal cages with limited water and light available, inadequate hygiene and poor diet, while they undergo years of repeated, traumatic, and non-sterile bile extraction^5^. The diet varies from human leftover food to swine feeds and to various mixtures of rice or soy mash while diets are rarely supplemented with vegetables or fruit. The farming practices and husbandry often result in irreversible health outcomes for the bears.

Hibernating free-ranging bears have been a bioinspiration due to their developed mechanisms that protect them against the manifestation of burden of lifestyle diseases that accumulate with age, such as muscle loss, osteoporosis, vascular disease, and chronic kidney disease (CKD)^6–11^. Hibernation as an evolutionary adaptation has generally equipped bears with the ability to modulate several metabolic pathways making them more resilient against organ damage and metabolic derangements^12^. Chronic low-grade sterile or bacterial hepatobiliary inflammation are the most common pathologies in bears rescued from bile farms^13,14^ and often accompanied by morbidities of other systems^14^. Whereas inflammation can be beneficial as an acute, transient immune response to harmful conditions, chronic inflammation has been posited to contribute to ageing processes, in part by the process of immuno-senescence and “inflammageing”^15,16^. Immuno-senescence is a dynamic process in which immune responses may be reduced, unchanged or increased in association with progressive cellular ageing and loss^17^. Inflammageing is defined as the chronic low-grade sterile inflammation that accompanies ageing^18^ and is essential in the aetiology and progression of age-related diseases, such as CKD^19^ often presented with multiple morbidities^20^. Several cellular and molecular mechanisms are involved in inflammageing, including cellular senescence, tissue hypoxia, mitochondrial dysfunction, defective autophagy and mitophagy, activation of the inflammasome, dysregulation of the ubiquitin–proteasome system and repeated host DNA damage response from dysbiosis (changes in the composition of the host microbiota)^21,22^. It has been hypothesized that chronic low-grade inflammation may have a critical role in initiating these changes that have the potential for accelerating the ageing process in humans^15,23^.

Premature ageing is observed in chronic diseases in humans and can manifest as muscle wasting, osteoporosis, frailty, obese sarcopenia, cognitive dysfunction and reduced renal and cardiovascular function^23,24^. Chronic inflammation has a major role in premature ageing leading to hardening of the blood vessels, development of atherosclerosis and increased cardiovascular risk of death^25^. Excess secretion of proinflammatory cytokines, often enhanced by gut dysbiosis and visceral obesity in combination with inflammasome activation from apoptotic cellular debris (immuno-senescence), are key features that play a role in hypertension and cardiovascular diseases, as well as diseases of the renal and musculoskeletal systems^26–28^.

In this study we present long-term effects of chronic bacterial and sterile hepatobiliary inflammation on the renal, cardiovascular, and musculoskeletal system in formerly bile-farmed bears in Vietnam. Through a biomimetic approach we investigated possible similarities with inflammation related to premature ageing in humans, and compared deviations to the healthy wild ursid phenotype.

## Results

At the first health examination all bears presented increased gallbladder wall thickness and were diagnosed with sterile or bacterial cholecystitis. More than half of the gallbladder aspirates (23/42) were positive for bacteria with most common isolates being *Enterococcus *spp., *Streptococcus spp* and *Escherichia coli*^13^. The majority of bears (N = 37) presented heterogeneous liver echogenicity, while some bears had blunted (N = 22) or mildly blunted liver edges (N = 13). The relationship between altered hepatic edges (mildly blunted, blunted) and presence of biliary infection was found to be significant (p < 0.001) and suggesting an association between liver fibrosis (see Table 5) and presence of biliary infection^29^.

Moreover, many bears presented clinical signs indicative of chronic diseases. Table 1 presents these clinical findings together with the number of affected individuals.

**Table 1:** Clinical findings from formerly bile-farmed bears indicative of chronic comorbidities.

Clinical findings	N affected/total (%)
Serum creatinine > 2.4 mg/dL	27/42 (64%)
Proteinuria	23/40 (58%)
Left ventricular hypertrophy > 1.2 cm	20/35 (57%)
Vascular retinopathy	13/38 (34%)
SAP > 200 mmHg (persistent)	12/42 (random)
Obesity	15/42 (36%)
Sarcopenia	12/42 (29%)
Obese sarcopenia	6/42 (14%)
Degenerative joint disease	15/42 (36%)

Model-based analysis including the group interactions at the different health check points for serum creatinine (CREA) and gallbladder wall thickness (GB wall) is provided in Tables 2 and 3, respectively. The predicted marginal means and standard errors for CREA and GB wall per each group and health check are given in Table 4.

**Table 2:** Coefficient estimates (standard errors) and p values for health check and different groups (best fitted linear mixed effect model) explaining the variations in serum creatinine.

Random effect	Std. error		
Individual bear	0.27		

Predictor	Coefficient estimate	Std. error	p value
(Intercept)	2.22	0.32	< 0.001
Liver echogenicity [homogenic]	− 0.26	0.55	0.64
Health check [2]	− 0.43	0.49	0.38
Health check [3]	1.99	0.73	0.01
Bactibilia [+]	0.04	0.4	0.92
Liver echogenicity [homogeneous] * Health check [2]	0.16	0.68	0.82
Liver echogenicity [homogeneous] * Health check [3]	− 2.51	0.87	< 0.001
Health check [2] * Bactibilia [+]	0.22	0.57	0.7
Health check [3] * Bactibilia [+]	1.28	0.68	0.06
AIC = 322.45

**Table 3:** Coefficient estimates (standard errors) and p values for health check and different bactibilia groups explaining the variations in gallbladder wall thickness.

Random effect	Std. error		
Individual bear	0.99		

Predictor	Coefficient estimate	Std. error	p value
(Intercept)	3.46	0.27	< 0.001
Bactibilia [+]	0.49	0.32	0.14
Health check [2]	− 0.83	0.26	< 0.001
Health check [3]	− 1.31	0.30	< 0.001
Bactibilia [+] * Health check [2]	0.37	0.37	0.33
Bactibilia [+] * Health check [3]	0.05	0.48	0.92
AIC = 309.30

**Table 4:** Predicted marginal means and standard errors of serum creatinine and gallbladder wall thickness per group and health check.

Variable (unit)	Group	Health check		
		HC 1	HC 2	HC 3
CREA (mg/dL)	Homogeneous	1.99 ± 0.5	1.82 ± 0.28	2.11 ± 0.3
	Heterogeneous	2.25 ± 0.2	1.92 ± 0.3	4.87 ± 0.6
	Bactibilia (−)	2.1 ± 0.33	1.74 ± 0.28	2.83 ± 0.4
	Bactibilia (+)	2.14 ± 0.35	2.01 ± 0.31	4.15 ± 0.49
GB wall (mm)	Bactibilia (−)	3.46 ± 0.27	2.62 ± 0.24	2.14 ± 0.26
	Bactibilia (+)	3.94 ± 0.24	3.48 ± 0.28	2.68 ± 0.38

**Figure 1:**
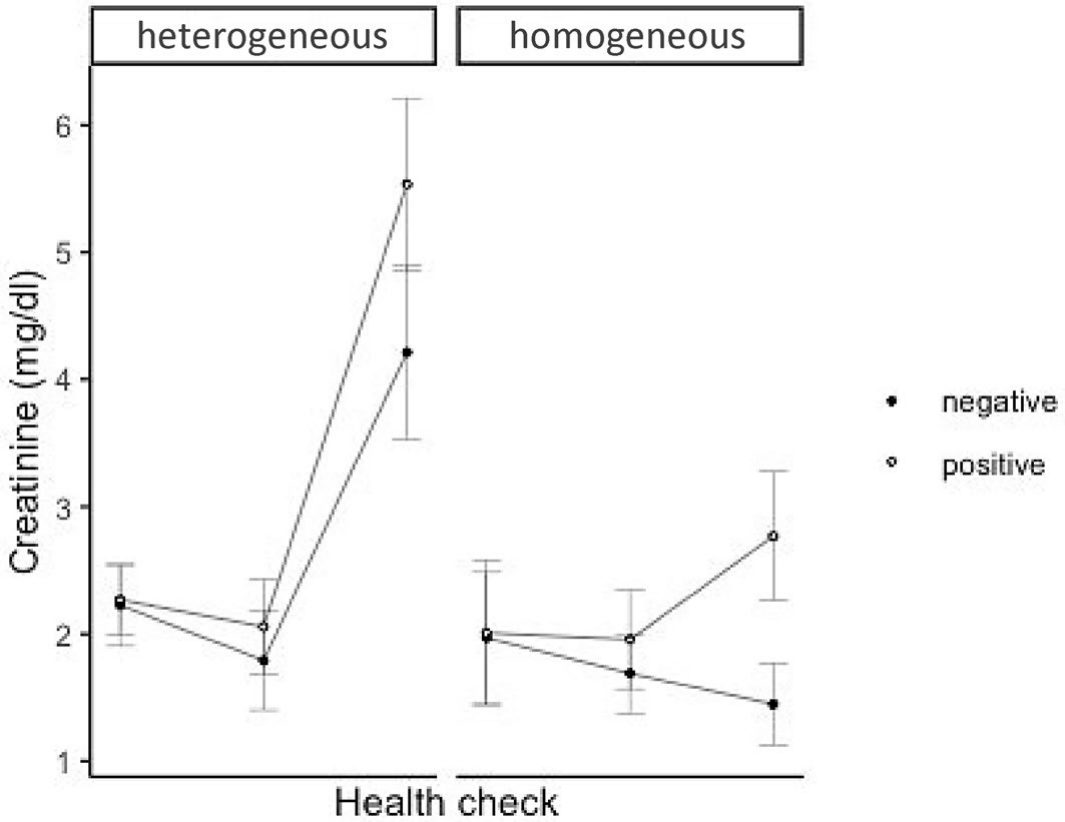
Effect of liver echogenicity (homogeneous, heterogeneous) and presence (positive) or absence (negative) of bacterial bile infection on levels of serum creatinine at the three health check points.

CREA was found to increase significantly at the third health check in response to persistent hepatic heterogeneous echogenicity and unresolved biliary infection with p values < 0.001 and equal to 0.02 respectively (Fig. 1, Table 4). In addition, CREA was significantly higher in bears with proteinuria (p = 0.02).

**Figure 2:**
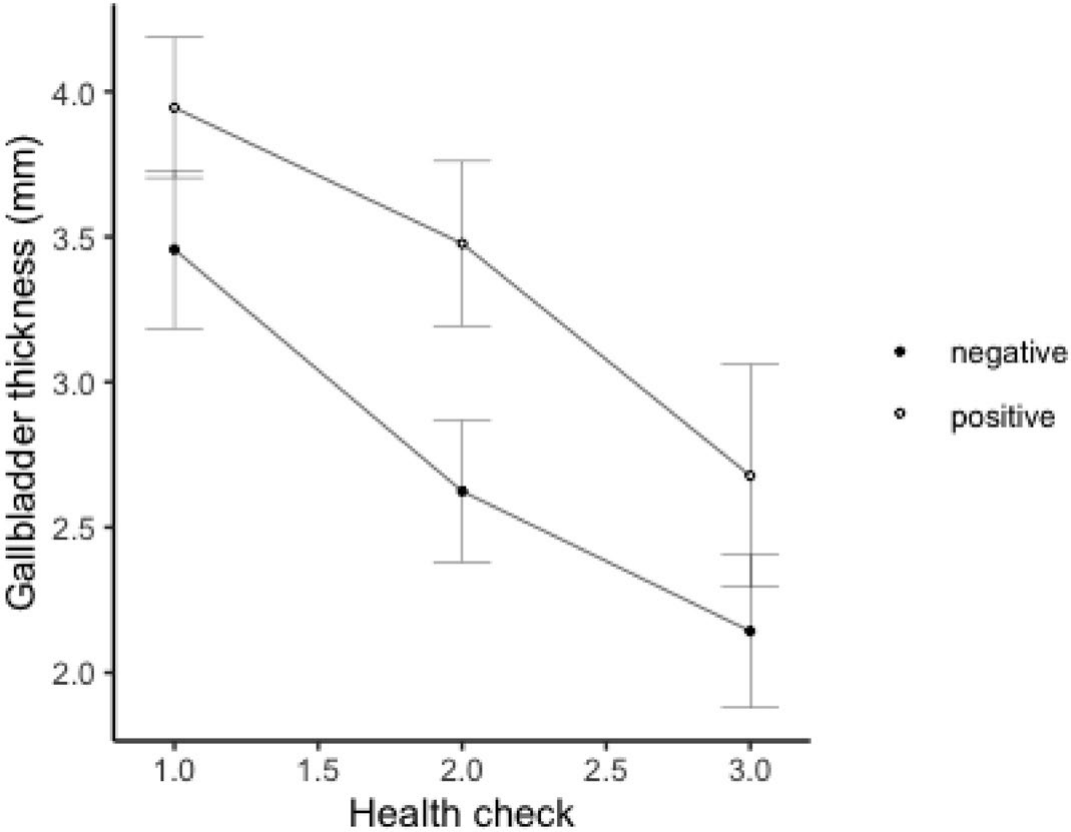
Effect of presence (positive) or absence (negative) of bacterial bile infection on the thickness of the gallbladder wall at the three health check points.

Furthermore, at the second health check, bears with unresolved biliary infection were found to have significantly thicker gallbladder walls in comparison to bears with negative bile cultures (p = 0.01, Fig. 2, Table 4). The gallbladder wall was also found to be significantly thicker in bears with retinal lesions (p < 0.001). No significant associations were identified between gallbladder wall thickness and the variables CREA, proteinuria, sarcopenia and obesity.

Sarcopenic bears had higher serum phosphate (mean = 5.9 ± SD = 1.8 mg/dL, p = 0.03) and sarcopenia was significantly associated with degenerative joint disease (p < 0.001) and obesity (p < 0.002). Obesity was also strongly associated with bactibilia (p < 0.001).

In comparison with active free-ranging brown bears^30^, bile-farmed bears had significantly higher C-reactive protein (CRP) levels (mean = 1.6 ± SD = 1.2 mg/L, p < 0.002).

## Discussion

We examined 42 Asiatic black bears that had been repeatedly extracted for bile over one decade. Our main finding was that all animals suffered from cholecystitis and more than half of them had positive bacterial bile cultures (bacterial cholecystitis). Cholecystitis in this cohort is believed to be a prolonged condition occurring after repeated episodes of mild cholecystitis (repeated traumatic bile extraction) and therefore characterized as chronic. Bacterial cholecystitis was associated with signs of hepatic fibrosis as well as with obese sarcopenia as compared to bears with sterile cholecystitis. In addition, unresolved bacterial cholecystitis was associated with elevated creatinine levels, a sign of CKD. Moreover, bile-farmed bears had significantly higher CRP levels in comparison with previously published measurements obtained from wild free-ranging brown bears^30^. While we did not have data from normal, age-matched Asiatic black bears for comparison, the overall frequencies of these conditions appear higher than expected for this cohort^6–11^.

In Vietnam bile is extracted through non-sterile, blind or ultrasound guided cholecystocentesis^13^. In addition to the risk for chronic bacterial infection, the procedure itself causes acute inflammation and production of prostaglandin E2 (PGE2) from the gallbladder wall^31^. Repeated acute inflammatory events related to gallbladder bile aspiration can become chronic through cytokine amplification and inflammatory cell recruitment^32^, that may result in gallbladder wall thickening and changes suggestive of hepatobiliary fibrosis. It is likely that repeated hepatobiliary trauma leads to inflammation and immuno-senescence that accelerate aging and the development of age-associated pathologies^15^. Indeed, signs of hepatic fibrosis were more prevalent in bears with active bacterial infection in the bile, than non-infected^29^ . In addition, after treatment (2nd health check) the bears that remained positive for bacterial species in their bile had significantly thicker gallbladder walls than the bears that responded to treatment. This may suggest greater inflammatory severity in the gallbladder^33^ in the presence of persistent bacterial infection. At the third health exam no significant differences between wall thickness are detected between the two groups, while inflammation tends to decrease in both groups (decrease in gallbladder wall thickness). We hypothesize that after treatment, the effect of the balanced diet and species-appropriate husbandry together with the discontinuation of bile extraction contributes to the overall health improvement of the bears and decrease of gallbladder inflammation. However, even though gallbladder wall thickness decreases over time in the majority of animals, it still remains above 1 mm (controls < 1 mm) suggesting ongoing low-grade inflammation or potentially permanent wall remodelling in response to chronic inflammation.

Furthermore, as serum creatinine increased in bears with persistent liver heterogeneous echogenicity and unresolved biliary infection, the systemic inflammatory component of hepatobiliary disease may promote fibrosis and reduced renal function by the following sequence of events. During chronic hepatobiliary inflammation pro-inflammatory cytokines promote cellular injury, induce non autonomous cellular senescence (i.e., bystander effects) and disrupt maintenance of the epigenetic machinery in many organs including the kidneys^34–36^. Blood monocytes then enter and proliferate into the injured kidneys resulting into macrophage accumulation in both the glomerular and tubulointerstitial compartment. The accumulated macrophages will differentiate into macrophage subsets, which can cause further renal injury and subsequent fibrosis^37,38^. Moreover, in obese bile-farmed bears we observed a significant correlation between bacterial cholecystitis and sarcopenia. In humans, sarcopenia and obesity are associated with a chronic proinflammatory state impeding metabolic processes and disrupting adipose and skeletal functionality (obese sarcopenia)^39^ that predicts poor outcome^40^.

Chronic stress during bile farming^41^ together with pro-inflammatory cytokines may promote hypertension^26,42,43^, as suggested by our findings of increased systolic blood pressure, proteinuria, increased left ventricular thickness and vascular retinopathy^44–46^. The association between hypertension and chronic inflammation is further suggested by our finding that bears with retinopathy have thicker gallbladder walls. Moreover, it can be hypothesized that the poor living conditions in captivity result in alteration of the gut microbiome^47^ and increased generation of Trimethylamine N-oxide (TMAO) predisposing to renal disease progression^7^. Cardiovascular disease in bile-farmed bears manifested as left ventricular hypertrophy and aortic dilation^48,49^ and may be derived from a combination of hypertension, inflammation, CKD, and disturbed microbiota. In this cohort, many bears presented with degenerative joint disease. The articular cartilage is especially sensitive to the presence of inflammasomes, resulting in chondro-senescence and degeneration^27^. Together with the lack of possibility for exercise in the cages, this likely accelerates joint disease progression in bile-farmed bears.

Chronic inflammation promotes muscle wasting via stimulation of protein catabolism and suppressed muscle synthesis^50^. Bears are physiologically equipped to tolerate prolonged periods of immobility during hibernation without developing muscle wasting^7,11,51^. In contrast to wild hibernating bears we found a high prevalence of sarcopenia in bile-farmed bears in response to years of immobility. High prevalence of hyperphosphatemia in sarcopenic bile-farmed bears was also found. Hyperphosphatemia is a driver of ageing and age-related diseases in mammals^2,52^ and together with chronic inflammation may contribute to loss of muscle mass and function. In analogy with our observation, high phosphate levels correlate with reduced renal function, poor and imbalanced diet, suboptimal living conditions, premature ageing, gut dysbiosis and inflammation in the general human population^52,53^. Moreover, in mice age-related loss of muscle mass and strength correlated with hyperphosphatemia. In addition, in cultured myoblast chemically induced hyperphosphatemia promoted cellular senescence, reduced their proliferative capacity and impaired myogenic differentiation indicating a possible mechanism involved in the development of sarcopenia^54^.

CRP levels were found to be significantly higher in the Asiatic black bears in comparison with wild free-ranging brown bears, which suggests ongoing inflammatory/infectious processes in the bile-farmed individuals^30^. In the human context elevated CRP is associated with inactivity, obesity, reduced renal function^55^ and increased cardiovascular risk^56^.

Bile-farmed bears may represent a valid animal model for studies of premature ageing processes and age-associated pathologies deriving from chronic low-grade inflammation, poor diet and suboptimal living conditions. Despite the remarkably evolved ursid metabolic plasticity, they deviate significantly from non-farmed bears. As such, the presented cohort may improve our understanding of the pathophysiology and detrimental effects of morbidities associated with poor captivity in animals and lifestyle related diseases in humans.

Even though treatment was administered to all bears, gallbladder infection and chronic hepatopathy could not resolve in all cases contributing to the development and progression of renal disease, while maintaining an inflammatory environment. This accords with a report of bears from Chinese bile farms, showing that liver enzymes and creatinine remained elevated up to 14 years post rescue^14^. This suggests that despite rehabilitation and attempts of treatment in some bears inflammation induced pathological changes persisted due to decreased capacity for repair of tissue dysfunction. Furthermore, years of bile extraction may overstimulate stress responses and natural immunity leading to a non-resolved state that augment the progression of age-related diseases^57,58^. In addition, we saw that treatment discontinuation may have also contributed to renal disease progression indicating that the deregulated immune function of the bears may require life-long therapeutic support.

It is acknowledged that the control data from healthy bears are from another bear species, although great differences are not expected within the *Ursidae* family. Additionally, data from normal age matched Asiatic black bears were lacking. Not all samples and measurements were collected from all bears, which limits the study partly. Specifically, CRP was measured in 9/42 bears, left ventricular thickness in 35/42 bears and blood pressure in 12 bears. Moreover, the retinas of 38/42 bears were examined, while 40/42 and 39/42 bears were sampled for urine and bile, respectively. Also, for the diagnosis of degenerative disease radiography was used as an imaging modality which has low sensitivity in diagnosing early stages of the disease. Finally, muscle and body condition evaluation were not objectively assessed due to limited technical availabilities in Vietnam, but rather based on visual and physical inspection by one and always the same expert.

## Conclusions

In bile-farmed Asiatic black bears chronic low-grade hepatobiliary inflammation in conjunction with poor husbandry and chronic stress seems to increase the risk for developing degenerative diseases, such as obese sarcopenia, CKD, and impaired cardiovascular function. The observed derangements could be considered signs of accelerated ageing in this cohort. The phenotype of bile-farmed bears contrasts markedly to the healthy phenotype reported in wild hibernating bears.

## Materials and methods

### Animals

Forty-two (24 female, 18 male) Asiatic black bears (*Ursus thibetanus*) aged between 12 and 17 years old (mean = 14.5) were rescued from private bile farms across Vietnam. The bears were transferred and permanently housed at BEAR SANCTUARY Ninh Binh (an animal welfare project by FOUR PAWS) in Northern Vietnam. All animals had been kept for bile extraction for at least 12 years with the exception of four confiscated bears that were farmed for 2–3 years. Routine health examinations including sampling, was performed in all 42 bears once, in 38/42 bears twice and in 23/42 three times.

After the first health exam, all bears received 60 days of oral choleretic treatment consisting of 5 mg/kg ursodeoxycholic acid (UDCA) BID, 15 mg/kg silymarin SID, 1000 mg of artichoke leaf extract SID and 1000 mg of *Curcuma comosa.* Additionally, bears with positive bacterial cultures received antibiogram specific antibiotics (with the exception of bacterial resistance) for 14 or 30 days^13^. Bile samples were obtained from 39 bears, with 20 of them sampled twice to assess antibiotic treatment efficacy. The second health exam was carried out after completion of the choleretic treatment and the third 6 months after the second. In total 132 blood, 59 bile and 40 urine samples were collected.

Five, non-bile farmed Asiatic black bears aged between 6 and 7 years old were regarded as clinically healthy and used as controls. The animals had within range biochemical and haematological parameters and were considered free of cholecystitis due to their thin (< 1 mm) anechoic gallbladder wall, the lack of wall oedema or adhesions and absence of sediment in the gallbladder. Moreover, their bile aspirate cultures were negative with no cytological evidence of inflammation or bacteria. In addition, retinal examination, urinalysis, thoracic radiographs and echocardiography was carried out in four out of five individuals and did not reveal any pathologies. The blood pressure was measured in two out of four controls during the first hour of anaesthesia and their systolic arterial pressure (SAP) ranged between 165 and 180 mmHg.

### Examination and sampling under anaesthesia

The bears were anesthetized by darting with a combination of 2.5 mg/kg ketamine (Ket-A-100^®^, Agrovet Market, Peru) + 0.05 mg/kg midazolam (midazolam 50 mg/mL, magistral formulation, Vienna, Austria) + 0.035 mg/kg medetomidine (medetomidine 20 mg/mL, magistral formulation, Vienna, Austria) + 0.05 mg/kg butorphanol (Alvegesic^®^ vet. 10 mg/mL, Vienna, Austria) intramuscularly or 2.2 mg/kg tiletamine-zolazepam (Zoletil 50^®^, Virbac, Carros, France) + 0.035 mg/kg medetomidine + 0.05 mg/kg butorphanol intramuscularly. In some bears (N = 12/42) intraarterial blood pressure (IntraTorr, IntraVitals, United Kingdom) was measured during anaesthesia at the femoral artery (*Arteria femoralis*).

Blood samples were collected from the jugular vein within the first 40 min post darting for complete blood count (mindray BC-2800 Vet AutoHematology Analyzer, China) and biochemistry (IDEXX VetTest 8008 Chemistry Analyzer, Germany). Serum creatinine (CREA) values > 2.4 mg/dL (211 µmol/L) were assumed to be elevated^59,60^ and indicative of CKD. CRP levels were measured by Gentian Canine CRP Immunoassay in nine bears.

Urine samples were obtained through ultrasound guided (MyLab™ One Vet-Esaote, The Netherlands) cystocentesis for urinalysis. Proteinuria was assessed via colorimetric reagent strip method with (−) meaning non-proteinuria, (± or +) mild proteinuria, (++) moderated proteinuria and equal or greater than (+++) as severe proteinuria. Specific gravity was measured with a refractometer. Furthermore, gallbladder bile was collected via percutaneous ultrasound guided (MyLab™ One Vet-Esaote, The Netherlands) cholecystocentesis with the bears in dorsal recumbency following clipping and aseptic preparation of their right upper abdominal quadrate^13^. The collected bile aspirates were submitted for bacterial culture and antibiogram^13^.

Animals with undetectable vertebrae and hip bones during palpation, obvious abdominal distention, absent waist and protruding abdominal fat were characterized as obese^61^. Moreover, bears with notable loss of muscle mass from the epaxial, gluteal, temporal and supra/infraspinous muscles were defined as sarcopenic. These bears were also observed in an awake state in their enclosures where they exhibited decreased muscle strength (i.e., difficulty and weakness during climbing). Obese sarcopenia was defined as obesity accompanied by significant muscle wasting. The body condition and muscle mass evaluation were performed during physical examination in all cases by the same experienced veterinarian (SK Kalogeropoulu). Additionally, the same examiner observed the mobility of these bears in an awake state.

One ventrodorsal, two laterolateral thoracic and radiographs of the skeletal system were performed to further assess the cardiovascular system and the condition of the joints, respectively.

Indirect ophthalmoscopy was carried out through a 20D condensing lens (VOLK^®^, USA) and the fundus of 28 individuals was examined.

### Hepatobiliary and cardiac ultrasonography

A convex 1–8 MHz probe (SC3421 VET; MyLab™ One Vet-Esaote, The Netherlands) was used to perform both hepatobiliary and cardiac ultrasonography.

The hepatobiliary tract of all 42 bears was evaluated with the animals in dorsal recumbency. The liver was examined for its echogenicity and edge features (sharp, mildly blunted, blunted). Mildly blunted and blunted liver edges (ultrasonographic features) have been found to correlate positively with liver fibrosis in humans with chronic hepatopathy^29^. Hepatopathy in the bears was characterized by hepatic heterogeneous echogenicity with or without altered edge features. Furthermore, the gallbladder was assessed for its wall echogenicity, presence of wall edema, adhesions and contents (i.e., sediment, sludge and gallstones). The gallbladder wall thickness was defined as the mean of the measurements obtained from the anterior, posterior, left lateral and right lateral gallbladder wall. Measurements greater than 1 mm were considered as increased wall thickness (compared to controls) and suggestive of gallbladder inflammation^33^.

Cardiac ultrasonography was performed in right lateral recumbency through the right parasternal window. The left ventricle was identified at the short axis view at the level of the papillary muscles. The left ventricle was assessed for its chamber size, contractility and its free wall was measured at the end of diastole. Left ventricular thickness was calculated as the mean value of the inferior, posterior and lateral free wall^62^. Measurements were obtained from 35 individuals and thickness greater than 1.2 cm was considered increased (compared to controls).

Table 5 provides information about the definition, diagnosis and further categorization of the variables assessed in this study.

**Table 5:** Definition of pathological conditions assessed in formerly bile-farmed bears, diagnostic method used (underlined) and findings, with further details on categorization.

Pathological condition	Diagnostic findings	Further categorization-comments
Cholecystitis	Ultrasound: increased gallbladder wall thickness (> 1 mm) +/− hyperechoic gallbladder wall, presence of sediment, adhesions Cholecystocentesis: bacterial culture and bile cytology	GB wall thickness → sensitive marker of GB inflammation^32^ *Bacterial*: positive bile culture (= bactibilia) *Sterile:* negative bile culture
Hepatopathy	Ultrasound: liver heterogeneous echogenicity with or without altered hepatic edges (mildly blunted, blunted)	Altered hepatic edges were considered a sign of fibrosis, a feature of chronic hepatopathy^28^
Hepatobiliary disease = cholecystitis + hepatopathy		
Retinopathy	Indirect ophthalmoscopy: hemorrhages, vascular spasms, retinal vessel aneurysms, and partial or total retinal detachment	Sign of hypertension
Left ventricular hypertrophy	Echocardiography: left ventricular thickness > 1.2 cm → Hypertrophy	Sign of hypertension
Proteinuria	Colorimetric reagent strip method: positive (+) for proteins (urine)	Sign of CKD and hypertension if proteinuria is from renal origin
Chronic kidney disease (CKD)	Blood chemistry: serum creatinine values > 2.4 mg/dL, (211 µmol/L)	Renal histology of five deceased bears with CREA greater than 2.4 mg/dL revealed interstitial fibrosis and inflammation
Hypertension	Blood pressure measurement: SAP > 200 mmHg (persistently) under anaesthesia	
Obesity	Palpation: undetectable vertebrae and hip bones during palpation, obvious abdominal distention, absent waist and protruding abdominal fat	
Sarcopenia	Palpation and observation: notable muscle loss from the epaxial, gluteal, temporal and supra/infraspinous muscles together with muscle strength decline	
Obese sarcopenia = obesity + significant muscle wasting		
Degenerative joint disease	Radiographs-skeletal system: joint space narrowing, osteophytes, articular surface cortical irregularity + /− sclerosis	

### Statistical analysis

All statistical analysis was performed using the software package R for Mac OS X/Windows^63^. Normality and homoscedacity of the model residuals were visually assesed by using qq plots and histograms.

The effects of hepatobiliary parameters and other explanatory variables on CREA over time were tested by using a linear mixed effect model (lmer package)^64^. Health check (1,2,3), liver echogenicity (homogeneous or heterogeneous), GB wall (mm), bactibilia (positive, negative) and sex were used as independent variables. It was hypothesized that CREA values will vary over time/health check between hepatobiliary variables and therefore their interactions were tested. To find the best subset of fixed effect, a model selection was performed by calculating Akaike's information criterion (AICc) values (dredge function, MuMin package)^65^. Changes in CREA at each health check between the groups of liver echogenicity and bactibilia were analyzed by using a post-hoc test (Tukey’s Honest Significant Difference test, lsmeans package)^66^.

To test for the effect of bactibila over time on GB wall thickness, another mixed effect model was computed (fixed factors: health check (1, 2, 3), bactibilia (positive, negative); random factors: individual bear; interactions: health check × bactibilia). The differences in GB wall thickness at each health check between bactibilia positive and bactibilia negative bears were assessed with a Tukey HSD test.

Moreover, a non-parametric Wilcoxon signed-rank test was used (model normality violation, non-repeated measurements) to evaluate the differences between the following comparisons: GB wall-retinopathy (presence, absence), CREA-proteinuria (presence, absence) and phosphate-sarcopenia (presence, absence).

For the comparison of the categorical variables bactibilia, liver edge features, sarcopenia, obesity, and degenerative joint disease a Pearson’s Chi-squared test followed by a Bonferroni correction was carried out. Specifically, the following comparisons took place: bactibilia-liver edge features, bactibilia-obesity, obesity-sarcopenia and sarcopenia-degenerative joint disease.

The CRP levels from bile-farmed bears were compared to free-ranging active brown bear (*Ursus arctos*) data (N = 16) as previously published^30^ using a One-Sample T-test.

For all statistical tests a p value < 0.05 was considered statistically significant.

### Ethical approval

All treatment and sampling were part of essential medical interventions, not related to experiments. All treatment and sampling protocols were reviewed and approved (written consent) by the institutional ethics and animal welfare committee of FOUR PAWS Viet (https://www.four-paws.org/campaigns-topics/sanctuaries/bear-sanctuary-ninh-binh). All methods were carried out in accordance with the guidelines for good scientific practice of FOUR PAWS International and the reported methods are in line with ARRIVE guidelines (https://arriveguidelines.org).

## Data Availability

The datasets generated and/or analysed during the current study are available from the corresponding author on reasonable request.
